# Autologous Bone Marrow Mononuclear Cells in Ischemic Cerebrovascular Accident Paves Way for Neurorestoration: A Case Report

**DOI:** 10.1155/2014/530239

**Published:** 2014-01-05

**Authors:** Alok Sharma, Hemangi Sane, Anjana Nagrajan, Nandini Gokulchandran, Prerna Badhe, Amruta Paranjape, Hema Biju

**Affiliations:** ^1^Department of Medical Services and Clinical Research, Neurogen, Brain and Spine Institute Private Limited, Surana Sethia Hospital and Research Centre, Suman Nagar, Sion Trombay Road, Chembur, Mumbai, Maharashtra 400071, India; ^2^Department of Research and Development, Neurogen, Brain and Spine Institute Private Limited, Surana Sethia Hospital and Research Centre, Suman Nagar, Sion Trombay Road, Chembur, Mumbai, Maharashtra 400071, India; ^3^Department of Neuro-Rehabilitation, Neurogen, Brain and Spine Institute Private Limited, Surana Sethia Hospital and Research Centre, Suman Nagar, Sion Trombay Road, Chembur, Mumbai, Maharashtra 400071, India

## Abstract

In response to acute ischemic stroke, large numbers of bone marrow stem cells mobilize spontaneously in peripheral blood that home onto the site of ischemia activating the penumbra. But with chronicity, the numbers of mobilized cells decrease, reducing the degree and rate of recovery. Cellular therapy has been explored as a new avenue to restore the repair process in the chronic stage. A 67-year-old Indian male with a chronic right middle cerebral artery ischemic stroke had residual left hemiparesis despite standard management. Recovery was slow and partial resulting in dependence to carry out activities of daily living. Our aim was to enhance the speed of recovery process by providing an increased number of stem cells to the site of injury. We administered autologous bone marrow mononuclear cells intrathecally alongwith rehabilitation and regular follow up. The striking fact was that the hand functions, which are the most challenging deficits, showed significant recovery. Functional Independence Measure scores and quality of life improved. This could be attributed to the neural tissue restoration. We hypothesize that cell therapy may be safe, novel and appealing treatment for chronic ischemic stroke. Further controlled trials are indicated to advance the concept of Neurorestoration.

## 1. Introduction

A stroke or cerebrovascular accident (CVA) is the rapid loss of brain function(s) due to disturbance in the blood supply to the brain tissue. It may be classified as ischemic (thrombotic or embolic) or hemorrhagic [1]. The clinical presentation corresponds to the area of brain damage. Ischemic stroke results from a loss of blood supply to a part of the brain, initiating the ischemic cascade [1, 2]. Brain tissue ceases to function if deprived of oxygen for more than 60 to 90 seconds which after approximately two to four hours leads to irreversible injury and infarction. The core area of infarction is called the “umbra” and the surrounding partially viable neuronal cortex is referred to as the “ischemic penumbra” [1, 2].

The mainstay in the management of ischemic stroke is early restoration of blood supply to the affected brain areas. This includes thrombolysis with agents such as tissue plasminogen activator (tPA), streptokinase, urokinase, and others [3]. These are effective modalities with limitations such as a short window period (three hours) for administration, narrow indications, contraindications, and complications. Despite early restitution, residual sensorimotor and cognitive-perceptual deficits are commonly seen which require rehabilitative management. Some neurological function recovers in these patients within the first three months, but only 40 to 60% of cases regain functional independence anytime between three months to ten years [4]. With most of the daily functions being dependent on hand functions, its recovery is crucial. It is seen that the recovery of the hemiplegic hand is often slow. Several mechanisms are presumed to determine recovery. These include the penumbral tissues, neural plasticity, rehabilitation, and behavioral compensation strategies [4].

As recovery of function is a slow and long drawn process and lacks definitive treatment, it becomes necessary to explore the possible options that may accentuate the restorative process. Cellular therapy has been reported to have beneficial therapeutic effects in stroke [5, 6]. The following case study aimed at observing the effects of intrathecal autologous bone marrow mononuclear cells (BMMNC) in chronic ischemic stroke.

## 2. Case Presentation

**Figure 1:**
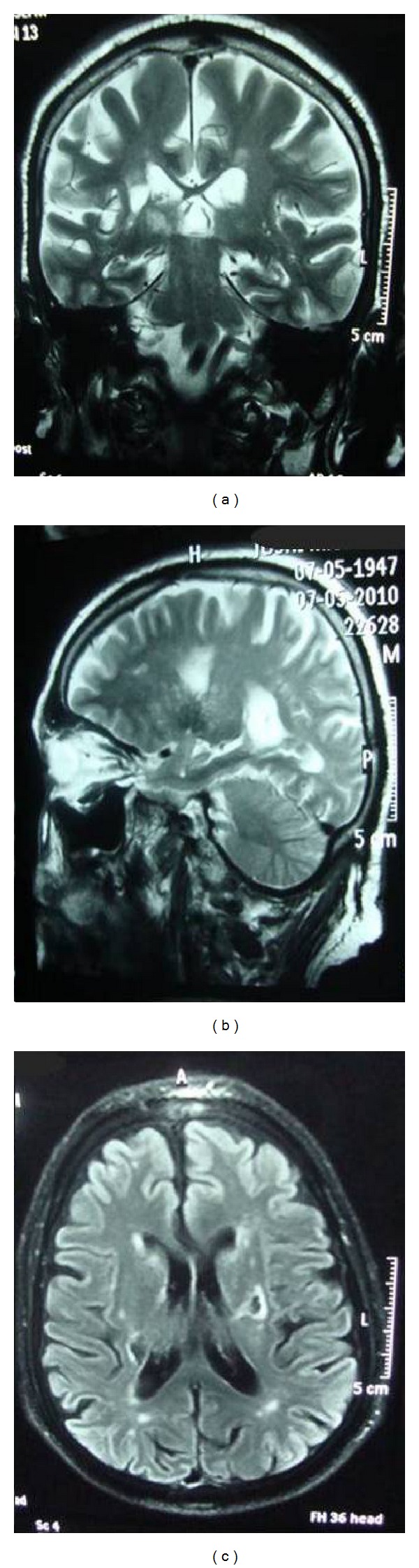
Magnetic resonance imaging of the brain; frontal, sagittal, and transverse sections showing an acute nonhemorrhagic right middle cerebral artery territory infarct with the putamen extending to the corona radiate, 12 hours after-stroke.

A 67-year-old male from Mumbai, India, developed sudden left-sided weakness with speech disturbance, approximately three and a half years back. During onset he was conscious, oriented, and ambulatory. His admission to a hospital was delayed by almost 12 hours after symptom onset, due to which thrombolytics could not be administered. On investigations magnetic resonance imaging (MRI) of the brain (Figure 1) showed an acute nonhemorrhagic right middle cerebral artery (MCA) territory infarct with the putamen extending to the corona radiata. There was no significant narrowing in the arteries of the circle of Willis. Chronic ischemic foci in the cerebral white matter, basal ganglia, thalami, and pons were noticed besides a chronic infarction of the corona radiata.

At nine months after-stroke and following physiotherapy the patient showed some recovery but had residual deficits. He was ambulatory with a cane and dependent for most of his ADLs. He was unable to use his left hand for his daily activities, perform isolated movements on the left side, open and close his fist, grasp objects, climb stairs, and so forth. Due to these limitations he sought to avail alternative treatment options. He came with increased tone in most muscle groups on the left side (grade 1 spasticity as measured on the Modified Ashworth Scale-M.A.S.). His voluntary control (graded based on Brunnstrom's stages of voluntary recovery after-stroke) was stage 1 to 2 in the left hand, stage 3 in left arm, and stage 3 in the left lower limb. Pain around the left shoulder had developed due to tightness of the flexor and adductor muscle groups. This resulted in limited, painful ranges of extension, abduction, and rotations. Both static and dynamic balance in sitting and standing were affected. His sensations and cognitive-perceptual functions were well preserved. Speech was slurred and fast indicative of mild dysarthria. His speech intelligibility was at level 2, that is, he required 1 to 2 repetitions.

Functionally, the patient was minimally dependant for his activities of daily living (ADL) as seen from his Functional Independence Measure (FIM) score of 109/126. He performed most activities one-handedly but preferred minimal assistance, whenever accessible. Performance of lower body dressing required moderate assistance. He had difficulty in bed mobility, transfers, and climbing stairs. He was using an aid (walking stick) for indoor ambulation. Outdoor mobility was limited and he required minimal support. Also, he experienced fatigue after walking for ten minutes or speaking continuously. His major concern was the absence of voluntary movements in the left hand, that is, his hand always remained partially fisted with thumb adducted and wrist in flexion. He was unable to reach out for, hold, or pick up objects with his left hand.

Considering his neurological and functional status nine months after-stroke, he was enrolled for intervention using intrathecal administration of autologous bone-marrow-derived stem cells followed by intensive rehabilitation. Detailed examination and assessments were conducted before intervention, at the time of discharge (i.e., one week after-stem cell administration) and at 3- and 6-month follow-up visits. In view of the improvements (described below) seen after the first intervention, he underwent the procedure for the second time 19 months after stroke. The patient was followed up at regular intervals for two years after intervention.

The patient was selected for intervention based on the inclusion criterion as per the World Medical Associations Helsinki declaration [7]. The treatment protocol is approved by the Institutional Committee for Stem Cell Research and Therapy (IC-SCRT). A signed informed consent from the patient was obtained. G-CSF (300 mcg) injections were administered subcutaneously, 48 hours and 24 hours prior to the bone marrow aspiration. Bone marrow (100 mL) was aspirated from the iliac bone under local anesthesia. A density gradient separation method was used to separate mononuclear cells (MNC). A viable count of the isolated MNCs was taken and checked by fluorescence-activated cell sorting (FACS) analysis. Percentage of CD34+ cells was identified using PE antibody. A total of 80 × 10^6^ cell were transplanted intrathecally and intramuscularly. Half of the MNCs fraction was then injected intrathecally into the L4-L5 level via a lumbar puncture using an 18-gauge Tuohy needle and catheter, over a period of 5–10 minutes. Intravenous methylprednisolone was administered (30 mg/kg over one hour) during the MNC transplantation. The remaining MNCs were then injected intramuscularly in various motor points.

The procedure was identical for both shots. During the second round of cellular therapy along with intrathecal administration, MNCs diluted in cerebrospinal fluid (CSF) obtained via lumbar puncture were injected intramuscularly into the motor points. A motor point is a point over the skin which corresponds to the entry of a motor nerve into the muscle where electrical stimulation (via electrode) causes contraction of the muscle. The motor points of the following muscles on the left side were injected with mononuclear cells: quadriceps, tibialis anterior, peronei, extensor digitorum, deltoid, extensor carpi radialis, extensor carpi ulnaris, triceps, lumbricals, opponens pollicis, and dorsal interossei.

Cellular therapy was followed by neurorehabilitation including regular physiotherapy, occupational therapy, and counseling to reduce his impairment and improve function. The voluntary control grading based on Brunnstrom's stages and FIM was repeated after the intervention and at every followup. These were used as outcome measures. The following changes were observed after the stem cell intervention and rehabilitation.

Clinical examination and functional assessments were analyzed to identify the neurological and functional changes in the patient. One week after cellular therapy and rehabilitation there was reduced intensity of pain and increased range of motion, both passive and active, in the left shoulder joint.

At the one-month follow-up assessment, static and dynamic balance were improved. He was able to rise from a chair easily and stand stable without support for 5 minutes. Lower body dressing was easier. Spasticity in the left upper and lower limbs decreased slightly. Hand grip was better and his ability to walk and climb stairs improved. Generalized fatigue was reduced. Improvement in speech was noted in terms of increased duration without fatigue.

At three months the above improvements were sustained and he was also able to walk indoors without the cane for 8 to 10 meters and commute independently using public transport. He was able to walk an outdoor distance of approximately 1500 meters in 20–25 minutes. His fatigue levels had further decreased.

At ten months, his functional status was maintained. Hand functions showed mild improvement. A second shot of cellular therapy was administered intrathecally and intramuscularly. Following the second round of cellular therapy, he showed improvements in the repertoire, control, and quality of left hand movements. Isolated movements of the elbow joint and abduction of the shoulder joint could be performed by him. He was able to bend down towards the floor and pick up a pen using a lateral pinch. Voluntary opening of the fingers made the release of gripped objects better (Brunnstrom's stage 4 of hand recovery). Active control was achieved in the movements of wrist extension, finger opening, and thumb abduction-adduction. Quality of gross grasp and release of large objects was better. His FIM score improved from 109 to 111. All the improvements persisted at the two-year follow-up visit.

## 3. Discussion

The incidence of CVA has greatly increased over the last decade. It is known to impact motor function and independence in ADL in a majority of the patients for a significant length of time. In this case, prior to the stem cell intervention some degree of recovery was noted but he was still dependant for his ADLs, causing major distress to the patient and his family. With the intervention and rehabilitation, we aimed at furthering and speeding the recovery process. We chose autologous BMMNCs as the mode of intervention because of the safety profile, absence of ethical issues, and easy availability in abundance. The intrathecal route of administration is a focused route which directly inserts the cells into the CSF. Thus, the accessibility of stem cells to the brain is enhanced. Intrathecal route is easy to administer and is devoid of any major side effects [8]. A second shot of intrathecal and intramuscular injections of MNCs was administered to further the improvements in hand functions. The intramuscular injections of MNCs help in the recovery of the weak muscles. The G-CSF administered before the transplantation helps in stimulation of CD34+ cells and also in the survival and multiplication of the stem cells [9].

Within an hour of an ischemic insult, there is a core area of infarction surrounded by an oligemic zone called the ischemic penumbra (IP) where autoregulation is ineffective. Cerebral blood flow to the penumbra is approximately 25% to 50% of the normal. It is said that reperfusion of these partially preserved areas within 2–4 hours after onset (“window period”) can partly or completely arrest and/or reverse the neurological deficits [3]. This area otherwise may have preserved cellular integrity and function for variable periods of time, making it a potentially salvageable area [1]. This patient missed the narrow “window period” for administration of tissue prothrombin activator. Due to this he was treated with standard medical and rehabilitative therapy. But even with this treatment, there was residual neurodeficit indicating incomplete functional recovery of neurons. Recently, bone marrow stem cell transplantation (BMSC) has been shown to have remarkable potential to repair the ischemia-damaged neural networks and form new synaptic connections [10]. The restoration of neuronal circuit activity likely contributes to better long-term recovery of sensorimotor function after BMSC transplantation [11]. We hypothesize that the administered mononuclear cells could have helped in the activation of the penumbral area. Earlier studies on BMSC transplantation have shown that directional migration of neuroblasts and their homing onto the injury site are enhanced [12, 13].

The transplanted cells secrete trophic factors and cytokines such as vascular endothelial growth factor (VEGF) and basic fibroblast growth factor (bFGF) that play a vital role in neovascularization. This neovascularization improves the oxygen supply to the site of injury which rejuvenates the marginally surviving cells or may enhance the local environmental factors that improve their function [11, 13]. Animal studies have exposed the various mechanisms through which BMSC exert their effects like increased expression of stromal cell-derived factor-1 (SDF-1) and brain-derived neurotrophic factor (BDNF) in the peri-infarct region, increased expression of the axonal growth associated protein-43 (GAP-43), and decreased axonal growth inhibiting proteins-ROCK II and NG2. In the peri-infarct area, they also improve survival of doublecortin (DCX) positive neuroblasts [13].

Following activation of these dormant neurons, the processes of neural plasticity, axonal sprouting, and neuronal reconnections may be accelerated by intensive rehabilitation including occupational therapy and physiotherapy [14].

The site of infarction in this patient was the territories of the middle cerebral artery in the right hemisphere. The middle cerebral artery supplies to the motor cortex (areas 4 and 6) wherein the representations of the arm, hand, and proximal lower limb are present. Focused motor improvements seen as reduced tone, decreased pain, increased ranges, and better voluntary control and quality of movements along with functional gains (pick up and release of objects and climbing stairs) correlate with the site of ischemia. This could indicate that the transplanted stem cells are driven to the site of injury. They probably replicate a neurorestorative mechanism similar to that which is achievable during the acute phase or “window period.” It has been shown that during the acute phase of injury after stroke, the inert bone-marrow-derived stem cells are mobilized and home onto the site of damage via the CXCR4/SDF-1 signaling pathway. But during the chronic phase, the quantity of mobilized bone-marrow-derived stem cells decreases tremendously [12]. Therefore provision of an increased number of stem cells has probably helped hasten and improve the recovery. Also, the rehabilitation interventions following cellular therapy seek to promote recovery and independence through neurofacilitation [15].

There were no short-or long-term adverse effects in this patient, two years after cellular therapy. The improvements in FIM scores, voluntary control, hand functions, and ADL independence can be attributed, though partially, to the cellular therapy.

The extent of injury and a correlation between clinical and investigative findings were not studied in detail for this case. Future studies could use imaging to address these factors for understanding the internal functional and structural changes rendered by the stem cells in view of the clinical findings.

## 4. Conclusion

We postulate that BMMNC along with rehabilitation may enhance the recovery in the chronic phase of ischemic stroke. The above reported case could be a beginning of a new paradigm for achieving neurorestoration in chronic stroke patients. It reestablishes the safety and viability of the intrathecal and intramuscular administration of autologous BMMNCs while indicating that more controlled, detailed, and systematic research in large group trials is required for greater applicability.
